# Recuperating Lung Decoction Attenuates the Oxidative Stress State of Chronic Obstructive Pulmonary Disease by Inhibiting the MAPK/AP-1 Signal Pathway and Regulating *γ*-GCS

**DOI:** 10.1155/2017/9264914

**Published:** 2017-03-20

**Authors:** Chunlei Li, Qi Shi, Yue Yan, Yanhua Kong, YanYan Meng, Tiezhu Wang, Xing Zhang, Haipeng Bao, Youlin Li

**Affiliations:** ^1^Beijing University of Chinese Medicine, Beijing 100029, China; ^2^The 2nd Pulmonary Department of TCM, The Key Institute of State Administration of Traditional Chinese Medicine (Pneumonopathy Chronic Cough and Dyspnea), Beijing Key Laboratory (No. BZ0321), China-Japan Friendship Hospital, Beijing 100029, China

## Abstract

*Purpose/Objective*. To evaluate the effects of Recuperating Lung Decoction (RLD) on the indices of oxidative stress in a rat model of COPD and detect the indices of the MAPK/AP-1/*γ*-GCS signal pathway for a further survey of the possible targeting site of RLD.* Methods/Materials*. The rats of COPD were treated with RLD. The protein levels of glutathione (GSH), oxidized glutathione (GSSG), 8-hydroxy-2-deoxyguanosine (8-OHdG), and 4-hydroxynonenal (4-HNE) were measured. In addition, the levels of key signaling molecules (extracellular signal-regulated kinases [ERK], the c-jun N-terminal kinase [JNKs signal pathway], and p38 MAP kinase [p38MAPK], AP-1 proteins [C-fos, C-jun], and *γ*-glutamyl-cysteine synthetase [*γ*-GCS-h]) of the MAPK/AP-1/*γ*-GCS-h signal pathway were assessed.* Results*. After treatment, the protein level of GSH and the ratio of GSH/GSSG were increased and the amounts of 8-OHdG and 4-HNE were decreased significantly in lung tissues when compared with the nontreated COPD group. Further results showed that the RLD could effectively inhibit the MAPK pathway by inactivation of p38MAPK and ERK and could also downregulate the AP-1 and the *γ*-GCS-h genes expressions in both protein and mRNA levels.* Conclusion*. RLD might improve the state of oxidative stress by downregulation of the expression of *γ*-GCS-h gene by inhibition of the MAPK/AP-1 pathway, thereafter enhancing the ability of antioxidation in COPD.

## 1. Introduction

Chronic obstructive pulmonary disease (COPD) is characterized by incompletely reversible airflow obstruction, inflammation, and emphysematous changes [1]. It becomes one of the four most important death-causing diseases in the world [2, 3], and its prevalence and death rate are increasing continuously [4]. Approximately 90% of the patients with COPD have a history of smoking, which is one of the causes of this disease [5]. In clinical practice, glucocorticoids, bronchodilators, and oxygen therapies are applied for reduction of the airway inflammation and for alleviation of the airway obstruction, thereby improving the quality of patients' lives [6, 7]. In present, many drugs are used for the treatment of COPD, while some of them such as steroids may cause side impacts. Therefore, there are increasing demands for herbal medicine, acupuncture, and other substitutive therapies on COPD in clinic [8, 9].

The imbalance of oxidation-antioxidant is one of the pathogeneses of COPD. Patients with COPD have higher oxidative stress levels in airway [10]. Oxidative stress promotes a combination of vascular endothelial cell growth factors with their receptors, which results in alveolar apoptosis and emphysema [11]. The human lung is continuously exposed to endogenous and exogenous oxidants, such as air pollution and smoking [12]. Relevant research studies indicate that the smoke of each cigarette contains more than 5,000 chemical compounds and 10^15^ free radicals [13, 14]. The MAPK signal pathway involves upregulation of the expression of antioxidant genes thereby enhancing the capability of damage resistance in cells [15]. The MAPK signal pathway is mainly made of the ERK, JNK, and p38MAPK. A large amount of oxidants could directly damage cells and tissues by stimulation of the phosphorylation of the ERK [16, 17], the P38MAPK [18], and the JNK. The phosphorylated ERK, JNK, and p38MAPK move to the nucleus to subsequently activate the expression of the transcription factor AP-1 [19, 20]. The interaction between the ROS and the tumor necrosis factor can also activate transcription factors NF-*κ*B [21] and AP-1 [22]. The AP-1 protein is an important transcription factor involved in regulating the expression of the *γ*-GCS gene. Extracellular stimulation activates the expression of AP-1 through a signal transduction pathway thereby enhancing the expression level of *γ*-GCS expression. The MAPK/AP-1/*γ*-GCS signal pathway induced by ROS plays an important role in the process and diagnosis of COPD and thus could pave a way for the therapy of COPD. It becomes very important in clinic to explore a drug that could significantly inhibit the signal transduction pathway to improve the imbalance of oxidation-antioxidation in COPD patients.

N-Acetylcysteine (NAC) is a mucolytic and antioxidant drug that may also influence several inflammatory pathways [23]. The increased GSH in lungs of COPD patients could antagonize the excessive oxidants. NAC is the precursor of intracellular-reduced GSH [24] and it could be transported into cells easily due to its small molecule. Therefore, NAC helps tissue cells tolerate damage caused by drugs and toxicants by the stimulation of the synthesis of GSH [25]. It has been shown that NAC reduces the release of thioredoxin (TRX) and glutaredoxin (Grx) and destabilizes the DNA binding activity of NF-*κ*B [26] and that it also affects p38MAPK, ERK, SAPK/JNK, AP-1 signal pathways [27].

Huangdi's Canon of Medicine, a classic book of traditional Chinese medicine, indicates that the human body is a unified organic whole, with holistic correlation among zang-fu organs. Development of chronic lung disease is closely related to lung and spleen functions. RLD is traditional Chinese medicine compound under holistic dialectical view, which regards lung and spleen as the core, affecting prognosis and outcome of COPD [28, 29]. Clinical application confirmed that theory of warming and tonifying lungs and spleen restores function of viscera and meridians during treatment of chronic lung diseases [30, 31]. RLD is the decoction, which based on the traditional Chinese medicine includes nine kinds of main medicinal plants (Table 1). These drugs are combined to strengthen functions of lung and spleen and to restore functions of viscera and meridians.

In our previous studies, we have constructed COPD rat models and established a scientific evaluation system [32]. RLD and its derived prescriptions can help scavenge oxygen-free radicals in serum of rats suffering from asthma and COPD [33]; it also improves inflammatory state of airway and lungs of rats with COPD [34]. This research aimed to uncover the biological function of the RLD in regulation of oxidation-antioxidant imbalance in COPD rats and found a possible molecular mechanism in inhibition of oxidative stress by detection of proteins involved in the MAPK/AP-1/*γ*-GCS signal pathway.

## 2. Methods

### 2.1. Plant Materials and Preparation of RLD

The nine herbal components of RLD were purchased from Tongrentang (Tongrentang Pharmaceutical Co., Ltd., Beijing, China) and meet the Chinese Pharmacopeia Standards 2016 upon testing (Table 1). The RLD premixed with water was decocted twice, followed by filtration and concentration (density is 1.25 at 60°C). Ethanol from the supernatant was collected. The extraction and preparation process above meet the pharmaceutical standards proposed by Beijing University of Chinese Medicine. The positive control drug NAC was purchased from (Fluimucil Co., Ltd., Hainan, China).

### 2.2. Animals, Diet

Male SD rats (4–6 weeks old) of SPF grade, with an average weight of 160 ± 10 g (certification of rat qualification number SCXK (Beijing), 2009-0007, Huafukang Technology Co., Ltd., Beijing, China), were raised in separate cages in a rearing room at 24 ± 2°C, with relative air humidity ranging from 45% to 65% under natural light and artificial light. The animals were allowed to eat and drink freely. Before the formal experiment, the adaptive feeding is adopted for one week. All the feeding conditions and the experimental processes conform to the internationally accepted US Principles of Experimental Animal Use (NIH publication number 85–23, revised in 1985). The experiment has been approved by the ethics committee of the China-Japan Friendship Hospital.

### 2.3. Experimental Methods

#### 2.3.1. Modeling

**Figure 1:**
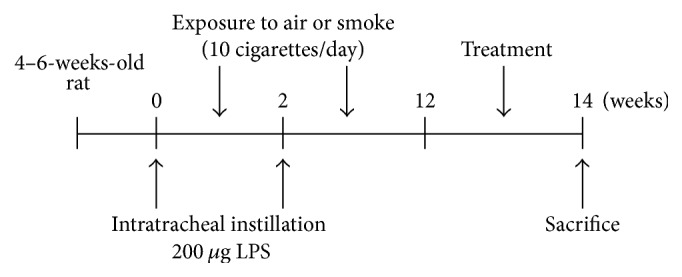
Experimental scheme diagram. The LPS was gradually dripped into the airway of male rats of SPF grade (4–6 weeks old) at week 0 and week 2. These rats were not exposed to smoke on the day of the surgery but were exposed to smoke for the remaining 12 weeks. From week 12 onward, administration was given continuously for two weeks and the rats were sacrificed and the samples were obtained at week 14.

The modeling method was based on our previous study with some modifications [35–37]. Briefly, 2% pentobarbital sodium with a final concentration of 3 mL/kg was injected into the abdominal cavity of the model group and the administration group (*n* = 8 for each group) for anesthesia at week 0 and week 2 of the experiment. After that, PBS solution (0.2 mL) containing 200 *μ*g of LPS (Sigma-Aldrich, St. Louis., MO, USA) was slowly dripped through the rat airway. As a control, 0.2 mL of PBS was dripped in the same way. On the day of each surgery (i.e., at week 0 and week 2), the rats of the surgery group were raised without smoke exposure, while for the rest of the days, the rats of the model group and the administration group were placed into a self-made smoke box (approximately 50 cm × 50 cm × 35 cm, made of organic glass, with a small door at the top center, four small air holes at sides, and a small smoking room holding cigarettes at the lower right corner; the smoke in the small room can flow into the dying box through the holes). The rats were exposed to passive smoking twice daily as follows: in the morning, five cigarettes (Daqianmen Cigarettes: tar yield 11 mg/cigarette, nicotine 0.8 mg/cigarette, CO yield 13 mg/cigarette, Shanghai Tobacco Group Co., Ltd. Shanghai, China) were lit simultaneously for 0.5 h for two times with a 10 minutes' interval; after that, all the experimental animals were placed back into the feeding box for normal foods and drinks. The second passive smoking was carried out in the afternoon in the same way. Meanwhile, the rats of the normal group were exposed to fresh air. The procedure was performed for 12 weeks (Figure 1). From week 12 onward, intragastric administration of RLD (3.65 g/kg) or N-acetylcysteine (0.5 g/kg), diluted with 3 mL PBS, was conducted. An equal quantity of normal saline was administered to the normal group. This process was performed for two weeks. Twenty-four hours after administration, the rats were sacrificed and bronchoalveolar lavage fluid (BALF) and lung tissue samples were collected.

#### 2.3.2. ELISA

Tracheal intubation was conducted after anesthesia into the rats, followed by three lavages with PBS. Then BALF was collected and centrifuged, and the supernatant was stored at −80°C. The concentrations of GSH, GSSG, 8-OHdG, and 4-HNE (R&D Systems, Minneapolis, MN) were measured in BALF by DAS-ELISA, according to the ELISA kits manufacturer's instructions.

#### 2.3.3. Immunohistochemistry

Slices were deparaffinized by Xylol, followed by rinse with phosphate-flushing fluid. After microwave-treated antigen retrieval, the samples were cooled below 35°C at room temperature. The slices were then incubated with 3% H_2_O_2_ at room temperature for 15 min. After that primary antibodies against P-P38 (1 : 100)/P-JNK (1 : 100)/C-fos (1 : 50)/*γ*-GCS-h (1 : 100) (Abcam, Cambridge, UK)/P-ERK (1 : 400)/C-jun (1 : 400) (Cell Signaling Technology, Boston, MA) were dripped into the samples and samples were incubated at 4°C for 24 h. After reheating at 37°C for 1 h, the respective secondary antibodies were added for incubation at 37°C. DAB coloring was then performed, and cell nucleus was redyed with Harris hematoxylin. The samples were dehydrated until becoming transparent and were sealed with neutral balsam. The slices were observed under a light microscope. Five different random views of each slice were observed. A semiquantitative analysis on integrated optical density (IOD) was conducted by HIS-IPP (Image Pro Plus 6.0) software.

#### 2.3.4. The Detection of MAPK/AP-1 by Western Blot

Right lung (40 mg) was cut off. Then, 400 *μ*L of CER I and 4 *μ*L of Halt™ Protease and Phosphatase Inhibitor Cocktail (Pierce Biotechnology Inc., Rockford, USA) homogenate were added to the lung tissue. The mixture was incubated on ice for 10 min followed by addition of precooled (4°C) CER II (22 *μ*L). The supernatant was collected by centrifugation for 10 min at 16000 rpm. Protein centration was measured by BCA method. Equal amounts of proteins were loaded in a 12% SDS-PAGE gel. After blotting onto PVDF membrane, samples were incubated with respective primary antibodies: phospho-ERK, C-jun (Cell Signaling Technology, Boston, MA), phospho-p38, phospho-JNK, ERK, p38, JNK, C-fos, *γ*-GCS-h (Abcam, Cambridge, UK), and *β*-actin (1 : 1000 dilution) and were incubated at 4°C overnight. Then, the respective secondary antibodies conjugated to HRP were incubated for 1 h, followed by three-time washing. The membrane was then incubated with ECL (Millipore, MA, USA) for luminescence generation. The image and the gray level were analyzed by Alpha Innotech and Adobe software. Protein expression level was evaluated by the *β*-actin ratio.

#### 2.3.5. RNA Extraction and Real-Time-PCR

A real-time PCR was carried out as follows to evaluate the effects of RLD on the AP-1 and *γ*-GCS in mRNA level in lung tissues. Total RNA was extracted (Qiagen, California, USA) from lung tissues (25 mg) stabilized by RNAlater (Qiagen). 2.5 *μ*g RNA was used as a template for cDNA synthesis by a reverse transcription (CDNA SuperScript®VILO™ cDNA Synthesis kit, ABI, California, USA). After a pretreatment by a select master mix (SYBR®Select Master mix, ABI), a real-time PCR was performed by the StepOne Plus Real-time PCR Systems (ABI). GADPH was used as the internal reference. The reaction conditions were set as follows: denaturation at 95°C for 2 min, 95°C for 15 sec, amplification at 60°C for 60 sec, 40 cycles. The Ct value was calculated as follows: ΔCT = purpose  Ct  (test  gene) − Ct  (GAPDH), ΔΔCT = ΔCT  (processed  sample) − ΔCt  (control  sample). The relative expression of the tested gene was calculated with the formula: Relative expression = 2^−ΔΔCT^ [38]. The test genes and primers are listed in Table 2.

#### 2.3.6. Data Analysis

The SPSS17.0 software (version 17.0, SPSS, Inc., Chicago, IL, USA) was adopted for statistics. All the measurement data were shown as the mean ± standard deviation (mean ± SD). The comparison among multiple groups satisfied the statistical principles. One-way ANOVA and Newman-Keuls test were used for intergroup analysis. If data did not conform to the normal distribution, the nonparametric Kruskal–Wallis test was adopted for a comparison; *P* < 0.05 indicates a statistical difference.

## 3. Results

### 3.1. Testing Results of Oxidative Stress Index in BALF

**Figure 2:**
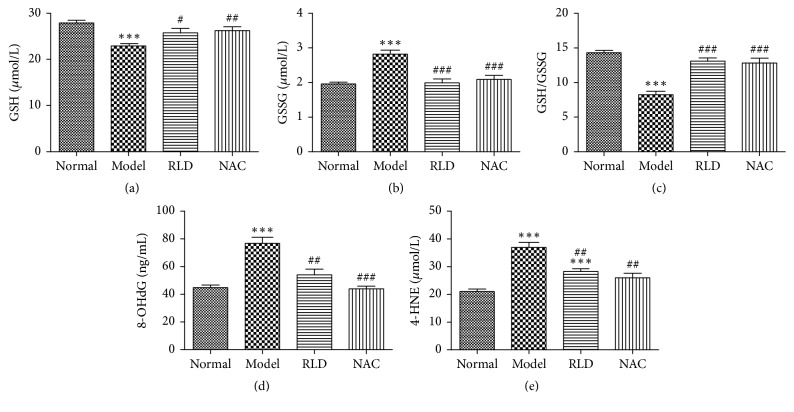
Influence of RLD on GSH, GSSG, 8-OHdG, and 4-HNE of COPD bronchoalveolar lavage fluid. (a) The GSH concentration of COPD rats was significantly lower than that of the normal group. The GSH concentration increased after NAC and RLD treatment. (b) The GSSG concentration of the COPD group was higher than that of the other groups. The GSSG level reduced after the NAC and RLD treatment. (c) The GSH/GSSG ratio after NAC and RLD treatment was higher than that of the COPD group. (d-e) The 8-OHdG and 4-HNE levels of the COPD group were higher than those of the normal group. The 8-OHdG and 4-HNE expression level reduced after NAC and RLD treatment. The data were expressed as mean ± SD. One-way ANOVA was adopted for statistical analysis. The Newman-Keuls was used for an intergroup comparison (^*∗∗∗*^*P* < 0.001 compared with the normal group, ^#^*P* < 0.05, ^##^*P* < 0.01, ^###^*P* < 0.001 compared with the model group; *n* = 8).

The GSH/GSSG is an important factor for evaluation of oxidative stress level and the 8-OHdG and 4-HNE are used to evaluate the oxidative damage of DNA and lipid peroxidation. In order to assess the function of RLD in regulation of oxidative stress, we measured the concentrations of the proteins involved in oxidative stress in the BALF liquid. The results showed that the GSH concentration of COPD rats was significantly lower than that of the normal group (*P* < 0.001) but increased after the RLD treatment (*P* < 0.05). In contrast, the GSSG concentration of the COPD group was higher than that of other groups, whereas its expression level was reduced after the RLD treatment (*P* < 0.001). Furthermore, the GSH/GSSG ratio after the RLD treatment was higher than that of the COPD group (*P* < 0.001). The 8-OHdG and 4-HNE expression levels of the COPD group were higher than those of the normal group but decreased after the RLD treatment (*P* < 0.01), although the 4-HNE level remained higher than that of the normal group. These results by RLD treatment above showed the same effects by NAC (Figure 2).

### 3.2. Detection of Expression Levels of Proteins Involved in the MAPK Signal Pathway in Lung Tissues by Immunohistochemical

**Figure 3:**
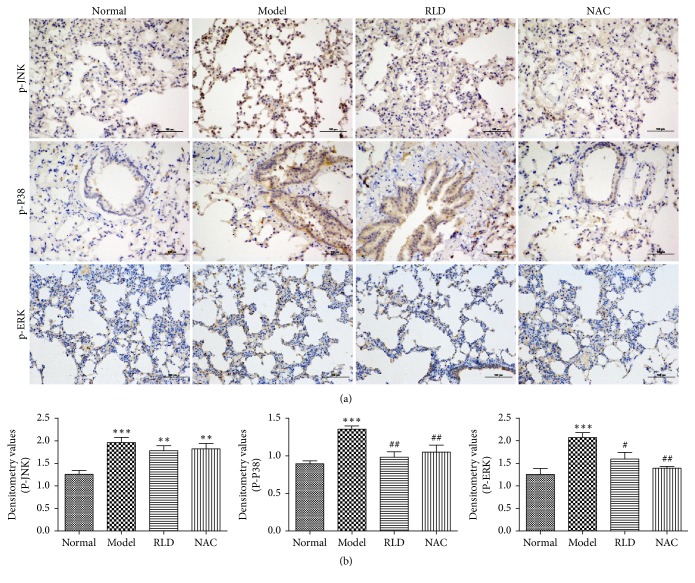
The phosphorylation levels of ERK, c-jun N-terminal kinase JNK, and p38MAPK are reduced after RLD treatment. (a) The expression levels and locations of p-ERK, p-JNK, and p-p38 in lung tissues were analyzed by immunohistochemistry. Brown positive expression was shown at the bronchial wall and alveolar septum. After RLD treatment, the positive expression of phosphorylation was reduced. (b) A semiquantitative analysis on p-ERK, p-JNK, and p-p38MAPK in lung tissues and an intergroup comparison were conducted. Stimulation of ROS significantly upregulates p38MAPK, ERK, and JNK phosphorylation. The RLD treatment effectively inhibited the phosphorylation levels of p38 MAPK and ERK. RLD showed no obvious influences on phosphorylation level of JNK. The data were expressed as mean ± SD. One-way ANOVA was adopted for statistical analysis. The Newman-Keuls was used for an intergroup comparison (^*∗∗*^*P* < 0.01, ^*∗∗∗*^*P* < 0.001 compared with the normal group, ^#^*P* < 0.05, ^##^*P* < 0.01 compared with the model group; *n* = 5). IOD, integrated optical density.

The ROS component in smoke exposure can activate all three MAPKs (ERK, JNK, and p38) [39]. To determine and evaluate whether RLD influences AP-1 and *γ*-GCS through the MAPK signal pathway, an immunohistochemical method was performed to detect the phosphorylation levels of the ERK, JNK, and p38 proteins in lung tissues. The results indicated that the oxidant significantly stimulated the phosphorylation levels of p38MAPK, ERK, and JNK, whereas these ascending phosphorylation levels were dramatically decreased after treatment by RLD suggesting that RLD could effectively inhibit the activation of p38MAPK (*P* < 0.01) and ERK (*P* < 0.05). RLD showed no obvious influences on the phosphorylation level of the JNK (Figure 3).

### 3.3. Detection of Phosphorylation Levels of MAPK Proteins in Lung Tissues by Western Blot

**Figure 4:**
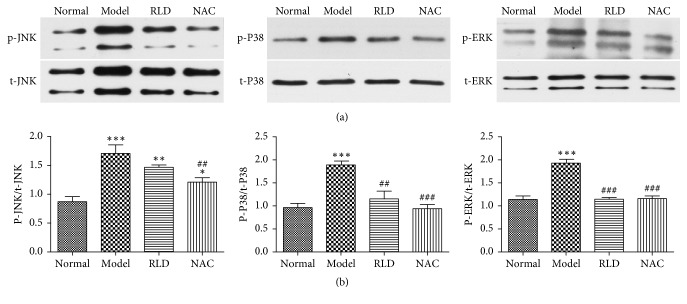
Phosphorylation levels of ERK, c-jun N-terminal kinase (JNK), and p38MAPK in lung tissues are reduced after RLD treatment. (a) Total ERK, JNK, p38MAPK, p-ERK, p-JNK, and p-p38MAPK protein amounts; (b) quantitative detection of ratios of p-p38MAPK/total p38MAPK, p-ERK/total ERK, and p-JNK/total JNK; P38 MAPK, JNK, and ERK in COPD significantly arose. Total expression levels of p38MAPK, ERK, and JNK were the same during the treatment. After RLD treatment, the phosphorylation levels of P38 MAPK and ERK were effectively inhibited. RLD showed no obvious influences on the phosphorylation level of the JNK protein. The data were expressed as mean ± SD. One-way ANOVA was adopted for statistical analysis. The Newman-Keuls was used for an intergroup comparison (^*∗*^*P* < 0.05, ^*∗∗*^*P* < 0.01, ^*∗∗∗*^*P* < 0.001 compared with the normal group, ^##^*P* < 0.01, ^###^*P* < 0.001 compared with the model group; *n* = 3, the blots represent at least four independent experiments, resp.).

To further determine and evaluate whether the RLD drug could influence AP-1 and *γ*-GCS through the MAPK signal pathway, we carried out a Western blot using phosphoric specific antibodies to test the phosphorylation levels of p38MAPK, ERK, and JNK. Total testing proteins amounts were determined to exclude any pipette errors that could cause the alterations of phosphorylation levels. The result showed that the phosphorylation levels of P38MAPK, JNK, and ERK in COPD were significantly increased (*P* < 0.001), whereas these enhanced phosphorylation levels could be dramatically inhibited by RLD. But total amounts of these test proteins were not changed during the RLD treatment. These results indicated that the phosphorylation levels of p38 MAPK and ERK could be stimulated by ROS but inhibited by RLD. RLD showed no obvious influences on the phosphorylation level of the JNK. These are in line with the results from our immunohistochemical experiments (Figure 4).

### 3.4. Expression of AP-1 in Lung Tissues

**Figure 5:**
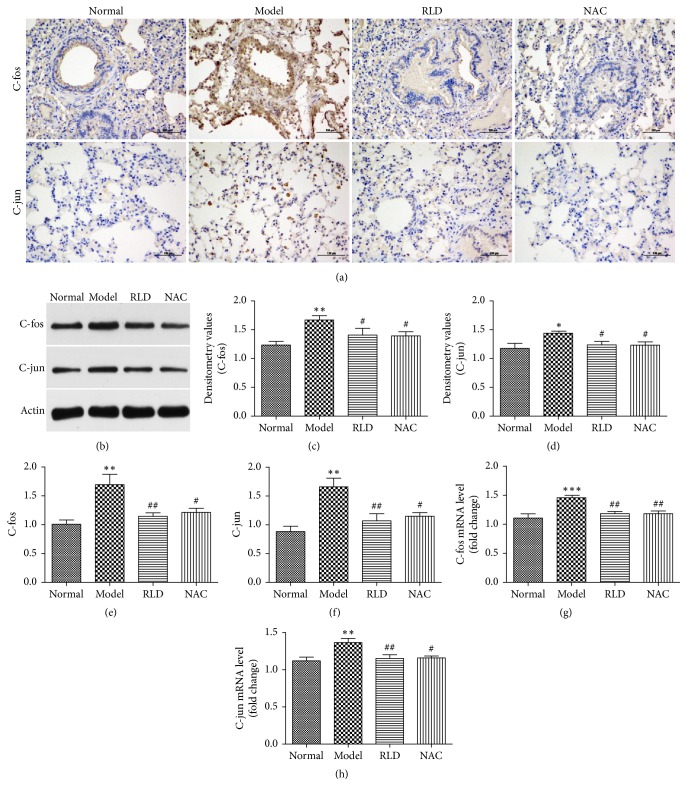
RLD reduces C-fos, C-jun protein, and mRNA expression in lung tissues. (a) C-fos and C-jun expressions and locations in lung tissues were analyzed by immunohistochemistry *n* = 5. Brown positive expression could be seen at the bronchial wall and alveolar septum. After the RLD and NAC treatment, the positive expressions of proteins were greatly reduced. (b) The C-fos and C-jun proteins expressions were analyzed by Western blot *n* = 3. Both proteins expressions in COPD group were increased. The expression was reduced after RLD treatment. (c) and (d) A semiquantitative analysis on the C-fos and C-jun level in lung tissues and an intergroup comparison were conducted by immunohistochemistry *n* = 5. (e) and (f) A quantitative analysis on the C-fos and C-jun protein in lung tissues and an intergroup comparison were performed by Western blot *n* = 3. The results were consistent with that from immunohistochemistry. (g) and (h) The C-fos and C-jun mRNA levels in tissues were tested by RT-PCR *n* = 3. The C-fos and C-jun mRNA levels in COPD group were increased but were reduced after RLD treatment. The data were expressed as mean ± SD. One-way ANOVA was adopted for statistical analysis. The Newman-Keuls was used for an intergroup comparison. (^*∗*^*P* < 0.05, ^*∗∗*^*P* < 0.01, ^*∗∗∗*^*P* < 0.001 compared with the normal group, ^#^*P* < 0.05, ^##^*P* < 0.01 compared with the model group; *n* ≥ 3).

The MAPK signal pathway induces the import and accumulation of the AP-1 into nucleus, thereby regulating the expression of target genes. To test whether RLD plays a role in regulation of AP-1, we first measured the expression levels of C-fos and C-jun proteins in lung tissues and compared them between different experimental groups by an immunohistochemistry technique. The data showed that both C-fos and C-jun proteins had higher expression levels around the alveolar septum and the airway in COPD group, while these ascending expression levels were reduced after RLD treatment (*P* < 0.05). These results were further confirmed by a parallel Western blot method. To further confirm these results, we quantified the mRNA levels of C-fos and C-jun in lung tissues by RT-PCR and found that both mRNA levels were increased in COPD group and were reduced after RLD (*P* < 0.05), suggesting that both C-fos and C-jun could be downregulated by RLD (Figure 5)

### 3.5. The Expression of *γ*-GCS-h in Lung Tissues

**Figure 6:**
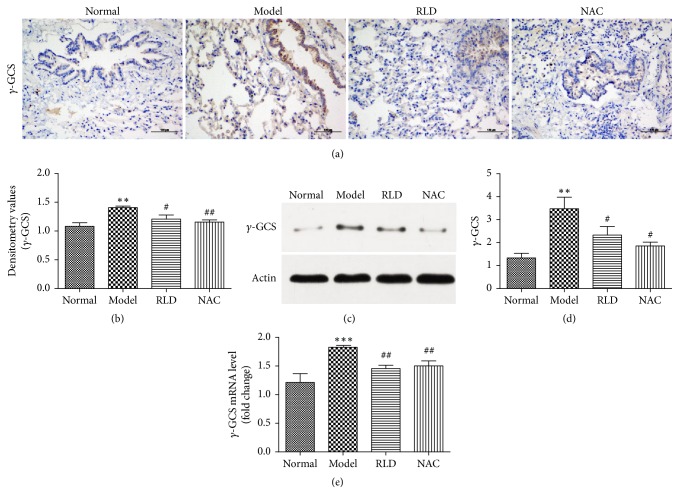
RLD reduces the expression of *γ*-GCS-h in lung tissues. (a-b) *γ*-GCS-h expression and location were analyzed by immunohistochemistry. Brown positive expression can be seen at the bronchial wall and alveolar septum. After RLD treatment, positive expression of protein was significantly reduced. (c) The *γ*-GCS-h proteins expressions were analyzed by Western blot. The proteins expressions in COPD group were increased. The expression was reduced after RLD treatment. (d) A quantitative analysis on the *γ*-GCS-h protein in lung tissues and an intergroup comparison were performed by Western blot. The results were consistent with that from immunohistochemistry. (e) *γ*-GCS-h mRNA level in tissues was tested by RT-PCR. The level of *γ*-GCS-h mRNA was higher in COPD group but lower after RLD treatment. The data were shown as mean ± SD. One-way ANOVA was adopted for statistical analysis. The Newman-Keuls was used for an intergroup comparison. (^*∗∗*^*P* < 0.01, ^*∗∗∗*^*P* < 0.001 compared with the normal group, ^#^*P* < 0.05, ^##^*P* < 0.01 compared with the model group; *n* ≥ 3).

The *γ*-GCS is the rate-limiting enzyme during the GSH synthesis process, and its activity directly influences GSH levels [40, 41]. In order to know whether the RLD affects the expression level of *γ*-GCS-h, we quantified the protein and mRNA levels of *γ*-GCS-h in lung tissues of different groups by immunohistochemistry, Western blot, and q-PCR, respectively. We found an increased expression of *γ*-GCS-h in both protein and mRNA levels on the tracheal wall and at the alveolar septum in COPD group and a reduced expression of *γ*-GCS-h in the group treated with RLD (*P* < 0.05). This suggests that RLD might play a role in downregulation of the *γ*-GCS-h protein (Figure 6).

## 4. Discussions

In this research, we successfully established a COPD rat model by the airway LPS dripping in combination with the continuous cigarette smoking. After treatment with RLD, we found that RLD might inhibit the MAPK/AP-1 signal pathway to regulate the expression level of *γ*-GCS, thereby reliving the oxidative stress of COPD.

The GSH-based oxidation-reduction system plays an essential role in maintenance of a dynamic balance of GSH/GSSG and becomes one of the most important antioxidant defense systems in maintenance of the physiological processes in pulmonary cells [42]. It also becomes an important factor in detection of the oxidative stress level. GSH functions as an important antioxidant barrier in the lung tissues in protection of cells against damage caused by oxidants. The 8-OHdG is a sensitive biomarker [43, 44] that is often used to evaluate DNA damage caused by ROS. 4-HNE, which is a product of oxidative damage, is a marker of lipid peroxidation [45, 46]. In this study, we observed a lower ratio of GSH/GSSG and increased levels of GSSG, 8-OHdG, and 4-HNE in BALF of our COPD rats. After treatment by RLD, we found that the ratio of GSH/GSSG was increased and the amounts of GSSG, 8-OHdG, and 4-HNE were reduced dramatically (Figure 2). These results above indicate that RLD could effectively improve the oxidative stress response in COPD rats and could show a similar function as the commercial antioxidant drug NAC for relief in COPD [23, 47].

During the process of COPD, oxides stimulate specific tyrosine kinases, such as epidermal growth factor receptors, and lead to autophosphorylation and activation of the tyrosine kinases [48], thereby stimulating the MAPK proteins by activation of the downstream proteins MEK-1 and Raf-1. They also could enhance the MAPK signal by inactivation of protein tyrosine phosphatases (PTPs) and could dissociate the complex of ASK-1 and TRX to further affect JNK/P38MAPK signal pathway of MAPK [49, 50].

In this research, we found that the phosphorylation levels of P38MAPK, ERK, and JNK in COPD rats were higher than those in normal groups from both immunohistochemical and Western blot experiments (Figures 3 and 4). These results may confirm that oxidants could activate MAPK signal pathways thereby causing phosphorylation of P38MAPK, ERK, and JNK, while RLD like the commercial drug NAC could effectively inhibit the phosphorylation of MAPK signal pathways.

An activated MAPK pathway of mitogen-activated protein kinase triggers the import and accumulation of AP-1 into nucleus, thereby regulating the target genes expression. It has been shown that MAPK activates JNK for translocation of the JNK from nucleus to the cytoplasm. The cytosolic JNK subsequently phosphorylates the Jun at sides Ser63 and Ser73 thereafter increasing the expression of AP-1. Therefore, the MAPK signal transduction pathway directly stimulates the AP-1 activity by increasing the amount of AP-1 [51]. The *γ*-GCS is the rate-limiting enzyme in the process of glutathione synthesis and its activity directly influences glutathione levels. The AP-1 plays an important role in regulation of the expression of *γ*-GCS; it has been reported that the expression of *γ*-GCS-h mRNA was regulated by the MAPK signal pathway in RAW264.7 mice macrophage cells, as inhibition of RKS/RAS/RAF-1/ERK/AP-1 abolished the DNA binding activity of AP-1 and gamma-GCS-h mRNA expression [52]. To evaluate the function of RLD on regulation of AP-1 and *γ*-GCS-h, we carried out RT-PCR, Western blot, and immunohistochemical methods to analyze both transcriptional and translational levels of C-jun, C-fos, and *γ*-GCS-h. The results above indicate RLD could effectively decrease the higher expression levels of C-jun, C-fos, and *γ*-GCS-h in COPD rats.

In traditional Chinese medicine, a mixture of Chinese herbal medicines is widely applied to the treatment of many pulmonary diseases [8, 53, 54]. RLD is made of nine major medicinal plants. The* Astragalus *polysaccharide (APS), an effective constituent in Radix Astragalus in RLD, plays some roles in antioxidation in lung tissues of rats with asthma by reduction of the phosphorylation level of the MAPK signal pathway to inhibit its excessive activation induced by OVA [55, 56]. The* cinnamaldehyde* present in Ramulus Cinnamomi significantly reduces the peroxidation of lipids and the amount of GSH by inhibition of the enzymatic activities of superoxide dismutase and by upregulation of the expression of Nrf2 [57, 58]. The* atractylon* in* Atractylodes macrocephala *Koidz was reported to have strong antioxidative abilities [59, 60]. The* Paeoniflorin* in Radix Paeoniae Alba targets the Nrf2/ARE signal pathway to inhibit the oxidative stress in the high-glucose-induced Schwann cells by reduction of the amounts of ROS and MDA and by the increased expressions of GST and GPX [61]. Fruits of Fructus Schisandrae Chinensis can improve the oxidative damage in AD by inhibition of RAGE/NF-*κ*B/MAPK and upregulation of HSP/Beclin [62]. We assumed that the synergistic effect of a mixture of medicinal plants might be more effective than a single herbal medicine on certain diseases which indicated that RLD might be a powerful therapeutic agent in reduction of the oxidative stress in COPD rats. In this study, we treated COPD rats with RLD and found that it could effectively stimulate the expression level of GSH and reduce the amounts of 8-OHdG and 4-HNE in lavage fluid, thereby attenuating the oxidative stress response in COPD. This therapeutic effect might be achieved by targeting the MAPK signal pathway to consequently reduce the expression levels of AP-1 and *γ*-GCS-h.

## 5. Conclusions

This study showed that RLD relieved the oxidative stress state of COPD, thereby achieving the treatment goal. These findings indicate that RLD might have a potential role in alleviation of the oxidative stress in airway, as well as in prevention and management of COPD. However, it still requires more investigations before the application of RLD to clinical therapy of COPD due to the highly delicate and complicated process of the MAPK/AP-1/*γ*-GCS signal transduction pathway.

## Figures and Tables

**Table 1:** The composition and amount of Recuperating Lung Decoction (RLD).

Herb name	Ratio	Amount (g)
Huang Qi (Radix Astragalus)	10	30 g
Fu Zi (*Aconitum carmichaelii* Debx.)	5	15 g
Gui Zhi (Ramulus Cinnamomi)	3	9 g
Bai Zhu (*Atractylodes macrocephala* Koidz)	2	6 g
Bai Shao (Radix Paeoniae Alba)	2	6 g
Wu Wei Zi (Fructus Schisandrae Chinensis)	2	6 g
Fang Feng (*Saposhnikovia divaricata* (Turcz.) Schischk)	2	6 g
Mai Dong (*Ophiopogon japonicus* (Linn. f.) Ker-Gawl)	2	6 g
Gan Cao (Glycyrrhizae Radix)	1	3 g
Total		87 g

**Table 2:** The sense and antisense primers sequences of *γ*-GCS, C-fos, C-jun, and GAPDH.

Gene name	Primer/probe sequences
GAPDH	5′- TTCCTACCCCCAATGTATCCG-3′
	5′-CATGAGGTCCACCACCCTGTT-3′
C-fos	5′-ATGATGTTCTCGGGTTTCAACG-3′
	5′-GGGATAAAGTTGGCACTAGAGACG-3′
C-jun	5′-AAACGACCTTCTACGACGATGC-3′
	5′-CGGTGTAGTGGTGATGTGCC-3′
*γ*-GCS-h	5′-GGAGGAACGATGTCCGAGTT-3′
	5′-CTGGAAAGAAGAGGGACTTAGATG-3′

*γ*-GCS-h: *γ*-glutamyl-cysteine synthetase.
